# Parental Experiences of Distance Learning in Families with and without an Adolescent with ADHD/ASD: A Large Qualitative Survey Study

**DOI:** 10.3390/ijerph21040388

**Published:** 2024-03-23

**Authors:** Lisa B. Thorell, Anna-Karin Klint Carlander, Youstina Demetry, Lisa Marainen, Sarah Nilsson, Charlotte Skoglund

**Affiliations:** 1 Department of Clinical Neuroscience, Karolinska Institutet, 17177 Stockholm, Sweden; youstina.demetry@ki.se (Y.D.); l.marainen@gmail.com (L.M.); nilsarson@gmail.com (S.N.); 2 Department of Clinical Sciences at Danderyd, Karolinska Institutet, 17177 Stockholm, Sweden; annakarin.carlander@icloud.com; 3 Department of Women and Child Health, Uppsala University, 75124 Uppsala, Sweden; lotta.borg_skoglund@uu.se

**Keywords:** COVID-19, distance learning, ADHD, Autism Spectrum Disorders, qualitative

## Abstract

One of the greatest COVID-19-related challenges for children and their families was managing distance learning due to school closures. We also know from previous research that families with a child with a neurodevelopmental disorder such as ADHD or ASD were struggling more than others but also experienced some positive effects. However, few qualitative studies have been conducted. The present study therefore aimed to investigate parental experiences of the negative and positive effects of distance learning during the COVID-19 pandemic in a large sample of families with an adolescent with ADHD and/or ASD and a matched comparison group (*n* = 682). Data were collected through open-ended questions as part of a larger survey study. Five main themes with different sub-themes were identified for both negative and positive effects: (1) Teaching, (2) Social, (3) Support, (4) Child factors, and (5) Home environment. In addition, the main theme “Technical problems” was identified for negative effects. Families with a child with ADHD/ASD reported negative effects related to “Child factors” and “Support” more frequently than the controls, as well as negative effects related to some aspects of “Teaching” and “Technical problems”. Regarding positive effects, significant group differences were primarily found for the theme “Child factors”. These findings are discussed both in terms of how to best prepare for possible future pandemics, but also of how to best provide educational support for children with ADHD and/or ASD when schools are open.

## 1. Introduction

The COVID-19 pandemic led to disruptions in the lives of adults and children worldwide. For families with school-age children, one of the greatest challenges was managing distance learning, as schools in most countries were closed [1,2]. However, several different factors have been presented to account for why some individuals experienced larger COVID-19 effects compared to others [3]. Personal vulnerability is one such factor, and previous research has shown that families with a child with neurodevelopmental disorders such as Attention Deficit Hyperactivity Disorder (ADHD) and/or Autism Spectrum Disorders (ASDs) found distance learning especially difficult. Interestingly, research has shown that families with a child with ADHD/ASD experienced not only more negative effects of distance learning for both children and parents [4,5,6,7,8,9] but also higher levels of self-reported positive effects [4,10]. However, the number of qualitative studies in this area is limited, especially studies of clinical samples. We therefore lack more detailed information on the psychosocial impact of school lockdowns during the recent pandemic. This information is important not only to be prepared for possible future pandemics, but also to better understand how to best provide support for children with ADHD and/or ASD now that schools have re-opened. It is well known from previous research that children with ADHD and/or ASD are at increased risk for poor academic achievement [11,12], including school absenteeism, school drop-out, or having to repeat a grade [13,14,15]. Thus, generating increased knowledge about the positive effects of distance learning may help us better understand how to adapt the school setting to the needs of students with neurodevelopmental disorders. The overall aim of the present study was, therefore, to obtain more detailed information about parental experiences of both positive and negative effects of distance learning during the COVID-19 pandemic in families with and without children with ADHD and/or ASD attending mainstream schools.

### 1.1. Distance Learning in Children with ADHD and/or ASD

ADHD and ASD are common neurodevelopmental disorders, with meta-analyses reporting worldwide pooled prevalence rates of 6.6–7.8% for ADHD [16] and 0.4–1.7% for ASD [17]. ADHD and ASD are highly comorbid disorders, and both disorders have been strongly linked to deficits in executive functioning such as working memory, inhibition, and cognitive flexibility [18,19], and these deficits have also been linked to academic underachievement [20,21]. Distance learning has most likely put extra high demands on executive functioning by placing increased expectations on adolescents to plan and organize learning activities. In addition, both ADHD and ASD are highly heritable disorders [22,23], which means that the proportion of parents with the same disorder or underlying neuropsychological deficits (e.g., executive function deficits) as their child is higher than the proportion in the general population. Previous research has also shown that parents have been largely responsible for children’s learning during distance learning [24]. Thus, it is hardly surprising that previous quantitative research has shown that families with children with ADHD and ASD have experienced more challenges with distance learning compared to families without children with mental health problems. This has included challenges related to increased mental health problems (e.g., stress and anxiety), child maltreatment, disruptions of daily routines, and lack of professional support [25,26]. However, as there are very few qualitative studies available, we lack in-depth knowledge about the challenges and potential benefits associated with distance learning in families with a child with ADHD and/or ASD.

### 1.2. Previous Qualitative Research on Effects of Distance Learning during the Pandemic

To our knowledge, only two prior qualitative studies have focused specifically on distance learning in families with a child or adolescent with ADHD, and no studies have looked at ASD. Roy et al. [27] investigated both challenges and benefits associated with distance learning as experienced by parents of children with and without ADHD aged 6–17 years. Regarding negative effects, they identified themes related to the child (i.e., the child has difficulties staying on task and lacks motivation), teaching (i.e., problems with remote instruction), social (i.e., lack of social interaction), and parental support (i.e., challenges juggling both teaching and work responsibilities). Positive effects were primarily related to increased family time, flexibility in schedule/learning, parental understanding of the child’s needs, and child independence. Surprisingly, some parents also reported lower, rather than higher, levels of stress and anxiety. Few differences were found between families with and without ADHD. However, families with children with ADHD more often reported that the child had difficulties staying on task and less often reported that the child missed the social interaction occurring in the regular school setting. Findings also highlighted that parents with a child with ADHD struggled more with organization and time management during distance learning than did controls.

Hatton et al. [28] used semi-structured interviews with nine participants (one child, one parent, and seven school professionals) to ascertain how students with ADHD were impacted by distance learning. They identified three main themes related to increased anxiety, change in social interactions, and academic impact. The third theme included both positive and negative effects. Regarding positive effects, the study reported that students were better able to manage their ADHD symptoms without being judged by others, they felt more relaxed in the home setting, and they benefited from a more flexible work schedule during distance learning. However, these positive effects were only present for students with an appropriate at-home learning environment and a supportive adult who could provide routines and motivation for completing academic tasks.

Regarding ADS, no qualitative studies have investigated distance learning specifically. However, Fridell et al. [29] studied pandemic effects more generally among parents of children with ASD, adults with ASD, adolescents with ASD, and representatives of ASD interest groups. They found some relevant themes related to education, showing that individuals with ASD experienced challenges with resisting distractions in the home setting, poor communication, lack of motivation, and increased demands on self-management. However, some participants also found that studying at home was more efficient compared to regular schooling, with distance learning having better structure and involving more regular feedback from teachers.

In addition to the studies described above, we have only found one qualitative study [30] of 15 girls with a range of behavioral and intellectual disabilities (including autism) and one qualitative study [31] focusing on distance learning in typically developing children. These two studies also identified social aspects, difficulties with virtual learning, and lack of support/communication with the school as important challenges. Interestingly, both studies also identified some benefits of distance learning. However, the most common benefit appeared to be getting away from the social pressure and stress that many students, with and without mental health problems or neurodiversity, experience in school. Thus, this could best be described as the absence of the negative effects of regular school rather than a benefit of distance learning.

### 1.3. Aim of the Present Study

The adverse effects of distance learning may have a long-term impact and contribute to increased psychosocial inequalities. However, equally important is any indication of potential positive effects of distance learning. As described above, few qualitative studies have focused on distance learning among children with ADHD and/or ASD, and available studies have either included small sample sizes or focused on a wide age range. Many previous studies have also been limited in that they have used retrospective reports rather than collecting data during school lockdowns. The present study therefore aimed to investigate parental experiences of the negative and positive effects of distance learning during the COVID-19 pandemic in a large sample of families with an adolescent with ADHD and/or ASD and a matched comparison group. Through this more in-depth investigation of parental experiences of distance learning during the COVID-19 pandemic, insights may be gained that can be used both when creating guidelines for future pandemics and when investigating the extent to which new learning techniques used during distance learning can be used as an important complement to traditional teaching. Children and adolescents with mental health problems may find it especially difficult to transition back to regular school settings following prolonged distance learning during the pandemic. Thus, to prevent long-term school absenteeism in this vulnerable group, it is important to retrieve more detailed information on both negative and positive experiences of distance learning.

## 2. Materials and Methods

### 2.1. Participants and Procedure

The present project collected data from a total of 369 Swedish families with a child with ADHD and/or ASD who were receiving distance learning due to school closure during the COVID-19 pandemic. As only high schools were closed in Sweden, only families with an adolescent aged 15–19 years could participate in the study. All families except 28 (7.6%) chose to answer the open-ended survey questions and were therefore included in the present study. This resulted in 341 families with a child with a neurodevelopmental disorder, of which 174 (51%) were diagnosed with ADHD, 57 (17%) were diagnosed with ASD, and 110 (32%) were diagnosed with both ADHD and ASD. A comparison group (*n* = 341), individually matched to the clinical group based on sex and age, was randomly selected from a larger sample of families with children without any known mental health problems. Thus, 682 families in total participated in the study. Descriptive data for the four groups are presented in Table 1. There were no significant group differences in the child’s age, child sex, and immigrant background. However, in line with the heritability of neurodevelopmental disorders [22,23] and the well-documented adverse psychosocial outcomes of ADHD and ASD [32,33], parental age and educational level were higher among parents in the comparison group. Data were collected from March to June 2020 using an online survey. Recruitment to both the ADHD and/or ASD groups and the comparison group was accomplished via social media (including Facebook groups for families with ADHD and/or ASD and contacts with patient organizations). The participants received information about the aims of the study and that they could withdraw from the study at any time before completing the questionnaire. They also provided their final consent to participate in the study before submitting their answers. No reimbursement was offered for participating in the study. At the time of data collection, the children had received distance learning for a period between two and fourteen weeks, M = 7.17 (SD = 1.93), with no significant differences between the four groups (see Table 1). The study was approved by the Swedish Ethical Review Authority.

### 2.2. Materials

The present study included two open-ended statements: (1) Describe the negative effects that you and your child have experienced due to distance learning; (2) Describe the positive effects that you and your child have experienced due to distance learning.

### 2.3. Analyses

The qualitative data were analyzed by LBT, LM, and SN using version 12 of the NVivo software and following the procedures for reflexive thematic analyses described by Braun and Clarke [34,35]. This includes the following steps: (1) data familiarization, (2) systematic data coding, (3) generating initial themes from coded and collated data, (4) developing and reviewing themes, (5) refining, defining, and naming themes, and (6) producing the report. Analyses were carried out inductively, without a pre-conceived theory or conceptual framework, and we used a semantic rather than a latent approach, which meant that we focused on presenting the content of the data as communicated by the participants, without attempting to identify hidden meanings or underlying assumptions [35]. The individuals coding the data were unaware of the group belonging, except when the parents revealed this in their answers. Once the themes had been identified, the coders received information about the group belonging of the statements and examined whether the same themes could be applied to the families with a child with a neurodevelopmental disorder and the comparison group, or whether different coding schemes should preferably be used for the clinical and non-clinical samples. In addition to the qualitative analyses, we used chi-square analyses to investigate differences between the four groups regarding how often the parents mentioned the different themes and subthemes.

## 3. Results

The results showed that the same themes were identified for the ADHD/ASD group and the comparison group. Importantly, however, this does not imply that the levels of negative or positive effects were the same, only that the same themes could be identified from the open-ended responses provided by the parents (i.e., there could be increased levels of, for example, inattention among both the ADHD/ASD group and the comparison group, although an increase from different baseline levels). As further reported below (see Section 3.3), there were also differences between the ADHD/ASD group and the comparison group regarding how often the different themes were mentioned. In the description below, each subtheme is illustrated by exact citations from the survey.

### 3.1. Negative Effects

The first open-ended question concerned the negative effects of distance learning. The following six themes were identified: teaching, child factors, support, social problems, home environment, and technical problems. Each theme also included three to six subthemes, which are described below, organized based on how frequently they were reported by the parents. Table 2 also shows an overview of themes and subthemes and examples of specific quotes from the parents. Overall, 6% of the parents stated that they did not think distance learning had any negative effects.

#### 3.1.1. Teaching

The most frequently reported theme was referred to as *Teaching*, and this theme was defined as any comments related to the teaching the child received from school or any academic work the child was involved in during distance learning. The most frequently reported subthemes were that the distance learning provided by the schools had poor *Structure and clarity* with regard to what the students were expected to do, and that distance learning placed too *High demands* on the child. Many parents also reported that the *Quality* of the teaching during the school closure was poor. The third subtheme related to teaching was *Practical subjects*, and comments classified into this theme were related to practical subjects for students studying vocational high school programs. This could also include comments related to practical assignments such as laboratory work for chemistry or physics class. The negative effect here was primarily related to the fact that these practical assignments could not be conducted during distance learning and that this would cause problems for the child later in life.

The sixth subtheme was *Variation*, and this theme included comments related to the fact that there was too little variation in the school assignments during distance learning, with too much work taking place alone and too many written assignments rather than oral tests or laboratory work. Finally, a few parents provided comments related to problems with *Exams or grades*. Some parents were concerned about the fact that national exams were canceled during the pandemic. They also reported that some teachers found it difficult to give students a high grade because cheating was possible during online exams. Parents were mostly concerned about the child receiving too low grades, but there were also concerns about too high grades (see Table 2). In total, 33% of parents mentioned negative effects related to the theme *Teaching*.

#### 3.1.2. Child Factors

The second most frequently reported theme was referred to as *Child factors*. This theme can be described as effects of distance learning related to the child’s feelings or behavior. Parents felt that their children had difficulties taking *Responsibility* or that they were missing certain *Skills* needed to successfully manage schooling from the home setting (e.g., getting up and dressed on time, planning schoolwork). Parents also provided comments related to the child’s low *Motivation/energy level* during distance learning. The next two subthemes were related to mental health. Parents reported increased levels of *depression/anxiety* or that the adolescent had difficulties paying *attention* during distance learning. Some parents also reported that the child had difficulties refraining from using *Digital media* when he/she was supposed to be studying.

Finally, a few parents mentioned other child-related problems that could not be categorized into the subthemes mentioned above, and these comments were classified into *Other child problems*. These comments were often related to mental health in general (i.e., not feeling good) or specific problems that were not reported often enough to be classified into a separate subtheme. For example, some parents reported that the child had executive functioning deficits. In total, as many as 31% of parents mentioned some sort of child problem when they were asked to describe negative effects of distance learning.

#### 3.1.3. Support

The theme *Support* included comments related to four different aspects of support: (1) teachers’ support for students, (2) parents’ support for students, (3) schools’ support for parents, and (4) extra-educational support. Regarding *Teachers’ support for students*, a relatively large number of parents reported that teachers were not available for support as much as the students wanted and that the digital format led to difficulties receiving immediate help. The parents also noted that it was more difficult for teachers to notice who needed help. Regarding *Parents’ support for children*, the biggest problem appeared to be that quite extensive parental support was needed and that parents, rather than the school, were mainly responsible for their child’s schooling. Moreover, many parents found it difficult to juggle being their child’s teacher and simultaneously working full-time. Regarding *Schools’ support for parents*, many parents felt they received too little information. Finally, parents of children with *Special educational needs (SEN)* mentioned that the child’s extra support was not given at all, or that it was too limited, during school closures. Some parents thought this was detrimental because their child needed more, not less, extra support during distance learning compared to regular classroom teaching. Altogether, 26% of parents mentioned lack of support as a negative effect of distance learning.

#### 3.1.4. Social Factors

The theme *Social* included three subthemes. The first two subthemes were both related to problems with social isolation; we distinguished between comments related to social isolation in *general* and comments related to *friends* specifically. Social problems also included comments related to the fact that *family* life was negatively affected by distance learning; these comments were not related to social isolation, but rather to increased family conflicts. Altogether, 18% of parents mentioned comments related to the theme *Social*.

#### 3.1.5. Home Environment

The theme *Home environment* included quite varying aspects that were classified into three different subthemes: (1) food, (2) exercise/fresh air, and (3) space. Regarding *Food*, some parents felt that it was expensive and/or time-consuming to provide lunch for their child every day, while also managing their own full-time work and helping the child with his/her schoolwork. Some parents also reported problems with the fact that distance learning had negative effects on the amount of *Exercise and fresh air* the child got. Comments related to food and exercise were often regarded as highly problematic, because this resulted in inattention and low energy to perform school tasks. Finally, some parents provided comments related to the fact that there was too little space or that the home environment was not calm enough for the child to conduct his/her studies. Altogether, 10% of parents reported some type of problem related to the theme *Home environment*.

#### 3.1.6. Technical Problems

The theme *Technical problems* primarily included comments related to problems with the *Internet connection* at home, with some families experiencing that the connection was only stable in some parts of the home and that the child therefore had to sit in, for example, the living room, where there were many distractions. Some families also experienced problems with the internet connection for the child’s classmates. Parents also reported that that they had problems with the *teaching platform* used during distance education or with the child’s *Computer*. However, altogether only 9% of parents spontaneously reported comments related to the theme *Technical problems*.

### 3.2. Positive Effects

Interestingly, almost the same themes identified for negative effects were also identified for positive effects (see Table 3). The exception was technical problems, which were not found in the case of positive effects. As further described below, the subthemes were also different for positive and negative effects. It should also be mentioned that in addition to the five themes described above, 11% of parents reported that they could not see any benefits of distance learning.

#### 3.2.1. Child Factors

Just as for negative effects, child factors were defined as effects that resulted from some aspect related to the child. This included *Inattention*, although as a positive effect, with some parents reporting that the child could concentrate better at home. Some parents also reported improved *Mental health* during school closures. Another positive aspect was that the child had *Higher attendance* during online teaching, either because it was possible to attend even when one would normally stay at home during regular schooling or because online teaching reduced some of the obstacles that prevent some children from attending school (e.g., getting up early and traveling to school). Finally, some parents reported that, owing to distance learning, the child had learned how to take more *Responsibility* for things and learned new *skills*. Altogether, 38% of parents reported positive effects that were classified as being related to the theme of child factors.

#### 3.2.2. Home Environment

The most commonly reported subtheme related to the theme *Home environment* was related to the fact that the child did not have to *Transport* him-/herself to school. Another positive aspect mentioned by a relatively large proportion of parents was that children were getting more *Sleep*. We also identified a subtheme, *General*, which concerned comments indicating that some aspects of the home environment positively affected the children’s ability to learn. For example, some parents reported that their child thought the home setting was calmer, more pleasant, or had better technical equipment. *Food* was also mentioned as a positive aspect. Finally, a few parents also mentioned that the child got more *exercise and fresh air* during homeschooling. Altogether, 36% of parents mentioned aspects related to the theme *Home environment* as a positive effect of distance learning.

#### 3.2.3. Teaching

The most frequently reported subtheme related to the theme *Teaching* was *Flexibility and independent needs*, which was defined as teaching that was more flexible compared to regular school and better adapted to individual needs (e.g., work pace). Many parents also reported that distance learning had better *Structure/efficiency*, and some parents described *new methods* that were used and that improved teaching. Interestingly, some parents also reported that the teaching had higher *quality*. Finally, a few parents commented on the fact that digital teaching could be a good *alternative for children with high school absenteeism*, and some were hoping that schools would be less rigid after the pandemic and offer digital teaching to children who have great problems attending regular school. Altogether, 15% of parents mentioned better teaching as a positive effect of distance learning.

#### 3.2.4. Social

Positive effects for the theme *Social* were primarily related to the fact that some parents reported that their children did not like to go to school and felt better when they could *Avoid their peers*. The second most frequently reported subtheme was *Family*, and this was related to increased connection within the family due to distance learning and the fact that many parents were working from home themselves. A few parents mentioned *virus safety* as one positive aspect of distance learning. They were afraid of catching the virus themselves and were happy that their child did not have to attend school and thereby increase the risk of being infected. Finally, a few parents, primarily those with a child with ASD, mentioned *digital interaction* as a positive aspect (i.e., their child was included more when students met online instead of meeting at someone’s home). Altogether, 14% of parents mentioned comments related to the theme *Social* as a positive effect of distance learning.

#### 3.2.5. Support

Support included three subthemes: (1) parental engagement, (2) support from parents, and (3) support from teachers. Regarding *Parental engagement*, some parents reported that they better understood how their child’s schoolwork was organized and how well the child was doing in school due to distance learning. As mentioned above when describing negative effects, many parents felt that supporting their child during distance learning was a burden. However, there were also a few parents who reported *Parent support* as a positive aspect, and these comments were mostly related to the fact that they as parents could provide individualized support for their child. Finally, a few parents reported that the teachers provided good support during distance learning. Altogether, 7% of parents mentioned comments related to the theme *Support* as a positive effect of distance learning.

### 3.3. Differences between Families with a Child with ADHD/ASD and a Comparison Group

**Figure 1:**
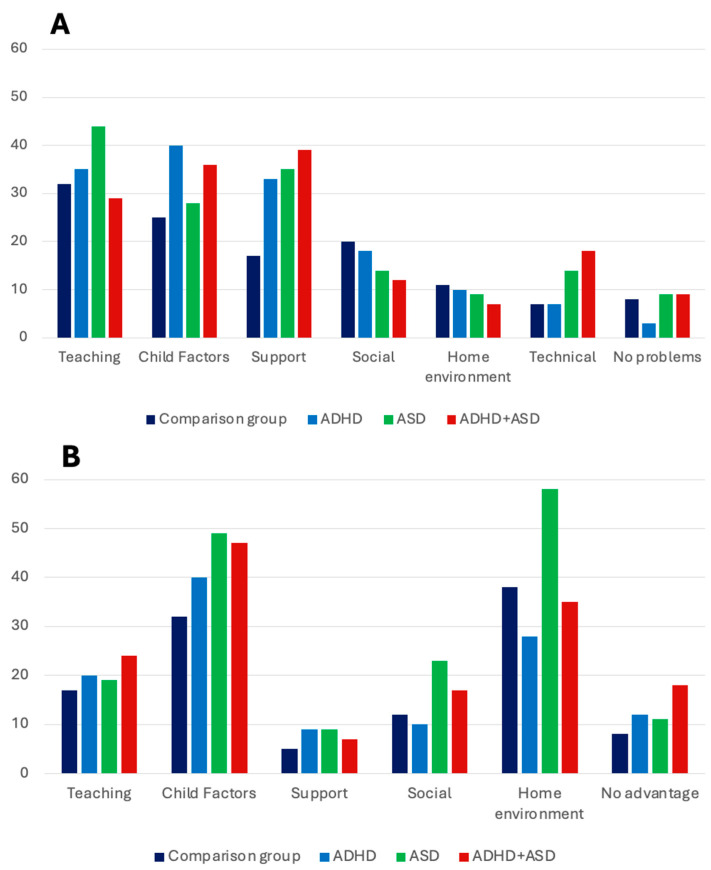
Group differences in the percentage of families reporting (**A**) negative effects and (**B**) positive effects of distance learning.

Finally, we examined differences between the comparison group and the three clinical groups regarding the percentage of families reporting problems in the different themes and subthemes. For negative effects (see Figure 1A), the results showed that there were no significant differences for the theme *Teaching* with 29–44% of the families reporting problems in this theme, all χ^2^ < 3.09. However, when investigating subthemes, results showed that the three clinical groups reported significantly more frequent problems with *Structure/clarity* (11–18%) compared to the comparison group (5%), all χ^2^ > 6.24, *p* < 0.05. Significant group differences were also found for the themes *Child factors*, with the ASD group and the ADHD + ASD group, but not the ADHD group, reporting negative effects related to child factors significantly more frequently than the comparison group, all χ^2^ > 12.99, *p* < 0.01. No significant group differences were found for *Home environment* and *Social*, all χ^2^ < 3.49. However, the ASD group (18%), but not the other two clinical groups, reported *Technical problems* more frequently than the comparison group (7%), χ^2^ = 11.73, *p* < 0.001. Finally, the comparison group (8%) reported no negative effects significantly more frequently than the ADHD group (3%), χ^2^ = 5.03, *p* < 0.05, but no differences were found to the other two clinical groups, all χ^2^ < 2.38.

For positive effects (see Figure 1B), the results showed at least one significant group difference in each theme, except for the theme *Support*. For *Child factors*, the comparison group (32%) reported these types of positive effects less frequently than both the ASD group (49%), χ^2^ = 6.37, *p* < 0.05, and the ADHD + ASD group (47%), χ^2^ = 7.53, *p* < 0.01. The difference was most pronounced for the subtheme *Higher attendance*, with 9–11% of children with ADHD or ADHD + ASD, compared to 3% of the comparison group, reporting higher attendance during distance learning compared to regular schooling. For attention and mental health, it was primarily the ASD group that reported positive effects. For the theme *Home Environment*, the ADHD group (28%) reported positive effects less frequently than the comparison group (38%), whereas the ASD group (58%) reported positive effects more frequently, both χ^2^ < 5.36, *p* < 0.05. For Teaching, significant group differences were only found for the subtheme *Alternative for children with school absenteeism*, with the three clinical groups (2–7%) reporting this more frequently than the comparison group (0.3%). For the theme *Social*, the ASD group (23%) more often experienced positive effects compared to the comparison group (12%), χ^2^ = 4.51, *p* < 0.05. No differences were found for the other two clinical groups. However, for the subtheme *Avoiding peers*, the ADHD group (7%), the ASD group (11%), and the ADHD + ASD group (15%) reported this positive effect much more frequently than the comparison group (1.5%), all χ^2^ > 10.64, *p* < 0.01. Finally, the ADHD + ASD group (18%) reported *No positive effects* more frequently than the comparison group (8%), χ^2^ = 10.2, *p* < 0.01.

## 4. Discussion

In this qualitative study of 341 families with a child with ADHD and/or ASD, we aimed to explore negative and positive experiences of distance learning due to school lockdowns during the COVID-19 pandemic. Our results revealed six major themes: (1) Teaching, (2) Child factors, (3) Support, (4) Social, (5) Technical problems, and (6) Home environment. All themes included subthemes displaying both positive and negative effects (except for the theme “Technical problems”, which only captured negative effects). The six themes were identified both in families with and in those without a child with ADHD and/or ASD, although with some differences between the groups regarding how frequently different themes or subthemes were mentioned. Families with a child with ADHD/ASD reported negative effects related to *Child factors* and *Support* more frequently than the controls, as well as negative effects related to some subthemes for *Teaching* and *Technical problems*. Regarding positive effects, significant group differences were found for *Child factors*, as well as for subthemes *Alternative for children with school absenteeism* and *Avoiding peers,* with children in the clinical groups experiencing more positive effects than those in the comparison group. For the theme *Home Environment*, results were mixed with the ADHD group reporting this positive effect less frequently than the comparison group and the ASD group reporting this effect more frequently.

### 4.1. Negative Effects of Distance Learning

Previous quantitative studies have shown that families with a child with ADHD/ASD experience more negative effects of distance learning compared to those without a neurodiverse child [4,5,6,7,8,9]. This finding has been explained by the fact that children with neurodevelopmental disorders, especially those with ADHD, have more academic difficulties compared to controls [11,12]. Both ADHD and ASD are also associated with executive deficits [18,19], and it has been shown that executive deficits have a major effect on how children can cope with distance learning, both among children with ADHD/ASD and those without mental health problems [9]. However, ADHD and ASD are highly heritable disorders [22,23]. This means that parents of children with ADHD/ASD may be more likely than controls to have mental health problems and poor executive skills, making them more vulnerable to negative effects and also less able to support their children than parents without children with ADHD/ASD.

The qualitative approach used in the present study allows us to provide information about what effects parents themselves found to be of most importance, without relying on specific questions posed in a quantitative study. Qualitative studies can also provide more details than can quantitative studies, which is important for designing effective support for children in the event of future pandemics, as well as for developing new teaching methods to better support children with special educational needs. Regarding specific themes, we showed that *Teaching* was the most frequently reported theme for both parents of a child with ADHD/ASD and controls with regard to negative effects. Parents were concerned that distance learning was too demanding, that the teachers failed to communicate their expectations and provide the structure and planning needed for the students to carry out distance learning successfully. Teaching during the pandemic meant a more passive learning approach with limited variation. Parents also expressed concerns that exams, practical assignments, and opportunities for discussion and interaction were limited, entailing the risk that their child may receive flawed grades or lack important skills for later education.

Regarding *Child factors*, parents expressed concerns that their child did not possess the skills essential for managing distance learning, lacking motivation to get started or finalize tasks or being distracted by digital media. Concerns were also raised regarding the child’s mental health, and parents of children with ADHD/ASD reported high levels of depressive symptoms and anxiety. Furthermore, many parents reported lack of, or insufficient, timely *Support* from teachers. The asynchrony of communication via emails or digital links further raised the need for parental engagement, and parents generally reported receiving too little information on what was expected of their child. In line with previous work [36], parents of children with special educational needs reported insufficient or non-existent support, despite the fact that their child needed more, not less, support during distance learning.

Negative effects for the theme *Social* were related to increased social isolation from peers or social isolation in general, as well as to conflicts within the family. Results from quantitative studies have shown that social isolation was the most frequently recognized problem during the pandemic, also in adolescents and young adults with ADHD [4]. Moreover, in the present study, we found that a substantial proportion of parents of students with ADHD (18%), ASD (14%), or both ADHD and ASD (12%) reported that their child felt socially isolated during distance learning. However, by using qualitative coding of free-text responses rather than responses to specific questions, we found that children in the three clinical groups, especially those with ADHD + ASD, seldom reported feeling socially isolated from friends. Instead, the clinical groups reported social isolation more generally (i.e., being restricted to the home setting). In summary, these findings further emphasize the need to not view students with ADHD and ASD as a heterogeneous group and to therefore provide support based on individual needs rather than on the extent of the child’s diagnosis. Regarding family conflicts, it was surprising that such a small proportion of families (0–3%) reported problems in this theme, as it is well-known that ADHD is associated with a range of risk factors related to the family environment [37], and these are likely to have been exacerbated by the additional strains caused by the pandemic. However, it should be noted that, compared to several other studies, we asked about negative effects of distance learning specifically and not of the pandemic in general.

For the theme *Home environment*, parents reported negative effects due to increased costs and time spent providing daily lunches during school days. Parents were concerned about increasing symptoms of inattention and distractibility as children were getting less exercise and fresh air and had to use an impractical or insufficient workplace at home. This is in line with previous research showing that increased physical activity is associated with decreased impairment of both ADHD and ASD [38,39]. Indeed Hatton et al. [28] reported that access to an outdoor garden for physical exercise during the COVID-19 pandemic led to better control of ADHD symptoms and less stress compared to during regular schooling, most likely because children could manage symptoms of hyperactivity and impulsivity without the risk of peer stigmatization at school.

Regarding the theme *Technical problems*, only a few parents reported problems. However, the ASD and ADHD + ASD groups reported higher problem levels within this domain, where having difficulty with the school’s digital platform was the most frequently reported problem. In Sweden, computers are provided by the school for almost all adolescents, and the participants in the present study should therefore have had relatively extensive previous experience with handling digital platforms. However, when practically all teaching is conducted digitally, this appears to have created problems for the students and especially for those with ASD, who are sensitive to changes in routines and to new systems that do not appear logical to them.

### 4.2. Positive Effects of Distance Learning

One interesting finding was that a large majority (78–92%) of parents reported at least some positive effects of distance learning. It is also interesting to note that the themes identified for positive and negative effects were the same, although with different subthemes. This indicates that each family has its own experience, with some families experiencing one aspect as problematic while another family has the opposite experience. Thus, support needs to be provided based on individual needs. For example, some children find it less stressful to work at their own pace, whereas other students find this stressful as it requires high executive functioning skills, which many children with ADHD and/or ASD lack [18,19]. It should also be acknowledged that it was not uncommon for parents to report both negative and positive effects in relation to the same theme. Thus, some parents said that social isolation from peers was a negative effect, while at the same time also reporting that the child showed less social anxiety because he/she did not have to face classmates in person.

One of the most striking findings of the present study was that a relatively large number of comments in response to the statement regarding positive effects did not mention the presence of a positive effect, but rather the absence of a negative effect. For example, the most common subtheme for social factors was “avoiding peers”, and this subtheme was much more common in the ADHD/ASD group than in the comparison group. This is in line with many previous studies showing that children with ADHD and ASD, more often than other children, have peer problems [40]. This could also be taken to be in line with previous quantitative studies, which showed a decrease in bullying during distance learning [41,42].

Two other positive effects reported by parents were sleep and food. During the past decade, there has been an increased interest in the effects of sleep and nutrition on mental health problems. In Sweden, where the present study was conducted, free school meals are provided for all students. This is generally seen as a positive thing, as it can lead to decreased social differences and increased help for families with low socioeconomic status, as all children get at least one nutritious meal a day. However, children with autism have an increased risk of specific food adversities [43], which could make it difficult to eat the food served at school. Thus, being able to choose your own food during distance learning may have increased quality of life and school performance, especially in those with ASD.

Previous research has shown that children with ADHD and/or ASD feel that they are not treated fairly within the regular school setting [44]. Indeed, some parents argued that distance learning may be a better way for their child to be assessed objectively and fairly by teachers. One important result for societal actors and policymakers is that some parents suggested that distance learning would be a feasible option for children with school absenteeism, arguing that schools should allow digital teaching even after the pandemic lockdowns. However, it should be noted that distance learning for only certain students may not have the same benefits as distance learning for all students during a school lockdown. For example, parents reported positive effects for the *Family*, allowing for more quality time during the day when the child still has energy and better emotional regulation than following a full day at school. They also reported positive effects for *Support*, as distance learning gave an increased understanding of how the child’s schoolwork was organized and how the child was doing in school, enabling individualized support and adaptations. However, after the pandemic, most parents will be working outside the home setting and, therefore, be unable to socialize with and support their child during the day. Parents of a child with ASD also mentioned that the digital interaction in distance learning was positive, because their child was more equally included in the group during distance learning, increasing his/her sense of belonging. However, if distance learning is offered only to certain children, this could instead create a sense of exclusion, as this type of adaptation would only be offered to students with disabilities of some sort. Given this, we do not wish to argue that distance learning could never be a good option for children with ADHD and/or ASD. However, we feel it is important to carefully consider the potential risks and benefits associated with this type of extra-educational support. As discussed further below, some form of hybrid models of teaching might therefore be the best option for some children.

### 4.3. Practical and Clinical Implications of the Findings

Regarding the implications of the current findings for teaching practices for children with ADHD/ASD, we like to argue that “hybrid models” of learning (i.e., making some parts available both online and in person) might be helpful for some children with ADHD and/or ASD. We feel that such an option should at least be considered for students with high school absenteeism, students who might otherwise simply stay at home and not attend school at all. However, even though the pandemic has increased the technical skills among teachers, hybrid learning is likely to pose challenges, and it should also be noted that the home is often a safe space for children with ADHD/ASD and “blurring the boundaries” between the school and home could be problematic [45]. Sigh et al. [46] argued that there are many different strengths and weaknesses of hybrid learning that need to be taken into consideration. This includes aspects related to the student such as mental health problems or neurodiversity, but hybrid learning can also cause increased stress for teachers and have technological challenges. We do not know of any studies post-pandemic which have evaluated hybrid learning models for neurodivergent students.

Another important finding of our study that has practical implications is that a substantial proportion of adolescents with ADHD and ASD appreciated that distance learning also meant not having to interact in person with their peers. We therefore think that programs aimed at reducing the stigma of ADHD and ASD and increasing acceptance for individual differences should be given high priority. Previous research has shown that social acceptance is an important intervention target for improving academic outcomes among adolescents with ADHD [47] and that there is a public stigma related to ADHD and ASD, also among professionals working within the school setting [48]. Finally, the present study shows great heterogeneity in the responses, also between different families with a child with a neurodevelopmental disorder. This emphasizes the need to not just focus on overt symptom levels but also take underlying neuropsychological deficits such as executive deficits and emotion dysregulation into account when assessing the needs of individuals with ADHD [19,49]. In fact, high levels of executive function deficits have been shown to have greater impact on the challenges of distance learning during COVID compared to having ADHD/ASD [9].

### 4.4. Strengths and Limitations

Very few qualitative studies including large samples of children with ADHD and/or ASD have been conducted, and the present study thereby provides an important complement to previous quantitative studies in this area of research. In contrast to previous qualitative studies, we included a relatively large sample size. This allowed us to investigate group differences between the comparison group and each of the three clinical groups. Another strength was that the data were collected during school lockdowns rather than relying on retrospective reports. The present study also had some limitations. First, only qualitative data from parents were included, and it would have been valuable to also collect data from the children themselves and teachers. Second, as is inherent to anonymous cross-sectional surveys, our study is limited to the use of a convenience sample based on invitations distributed through social media. Consequently, we lack more detailed information about the characteristics of the population from which our sample was obtained. Third, families with low educational levels and an immigrant background were underrepresented in our sample. Fourth, the data were collected during the first phase of the pandemic, so it is possible that the negative effects of school closures increased over time. Fifth, the present study was conducted in Sweden, which imposed less severe restrictions during the pandemic compared to many other countries. In addition, Sweden is well-known for having a very high level of digitalization. Almost all high school students are given a computer by their school, and practically all families have a private high-speed internet connection in their home. Thus, Swedish schools were most likely better prepared for making the transition to online teaching compared to schools in other countries, and it is therefore unclear to what extent our findings can be generalized to other countries. Finally, it should be noted that the present study only included adolescents. The results, therefore, cannot be generalized to samples of younger children, especially as previous research has shown that the challenges associated with distance learning decrease with age [24], although not as much for those with ADHD/ASD compared to those without [9].

## 5. Conclusions

In conclusion, the present study provides further support that many families with a child with a neurodevelopmental disorder found distance learning during the COVID-19 pandemic especially challenging. We present several points that should be taken into consideration during possible future school closures and aspects such as stigma. The positive aspects of distance learning for neurodiverse children should be highlighted and taken into consideration during traditional education as well. However, the large number of themes and sub-themes that were identified also illustrate that experiences vary greatly between families and that individual needs should be taken into consideration rather than focusing solely on whether or not the child has ADHD and/or ASD.

## Figures and Tables

**Table 1:** Results of ANOVAs and chi-square tests examining group differences in background variables.

	Comparison Group (A) *n* = 341	ADHD Group (B) *n* = 174	ASD Group (C) *n* = 57	ADHD + ASD Group (D) *n* = 110	*F/chi2* (η^p^/V)	Post Hoc
Background variables						
Child age, M (SD)	16.81 (1.43)	16.70 (1.50)	16.84 (1.45)	16.99 (1.27)	1.02 ns (<0.01)	
Child sex, % boys	60.0	60.4	54.8	61.7	5.58, ns (0.06)	
Ethnicity, (%) foreign background	4.2	3.2	1.6	1.8	2.15, ns (0.05)	
Parent age, M (SD)	49.15 (4.18)	48.11 (5.62)	48.56 (5.69)	47.68 (6.32)	3.28 * (0.01)	A > B, D
Parent sex, n (%) mothers	85.4	91.7	87.1	91.3	17.06 * (0.10)	A < B, C, D
Parent education					18.97 ** (0.11)	A > B, C, D
Mandatory schooling only	0.0	1.0	1.6	0.0		
Completed secondary school	12.0	17.8	24.2	23.5		
University education	88.0	81.2	74.2	76.5		
Distance learning (weeks)	6.99 (1.90)	6.98 (2.18)	6.56 (2.28)	7.33 (1.97)	2.02, ns (<0.01)	

* *p* < 0.05, ** *p* < 0.01

**Table 2:** Results of the study for positive effects showing themes, subthemes, and quotes.

Themes/Subthemes	Quotes
**Teaching (33%)**	
Structure/clarity	*Unclear tasks, bad planning, and no structure* (Mother of boy, 17 years; ADHD)
High demands	*All teachers give the students large assignments to work with on their own—much higher workload for the students compared to usual* (Mother of girl, 16 years; ADHD)
Quality	*Bad communication between teachers so they all do things differently, which really leads to poor quality teaching* (Father of girl, 18 years; no diagnosis)
Practical subjects	*There is no practical work done at school now and I do not think he will acquire enough skills to get a job after high school* (Mother of boy, 18 years; ADHD)
Variation	*Fewer opportunities for discussions, fewer chances to share his thoughts with others. Instead, more passive listening and assignments that need to be done on his own after class* (Mother of boy, 18 years; ADHD)
Exam/grades	*It appears as if it is more difficult to get a higher grade than last semester just because the national exams where canceled* (Mother of girl, 16 years; no diagnosis) *Some teachers feel sorry for the students because of the bad quality teaching during the pandemic so they give them easier exams. I worry that my child will not have enough knowledge when entering high school next year* (Mother of boy, 16 years; no diagnosis)
**Child factors (31%)**	
Responsibilities/skills	*He does not take responsibility to dress himself before the online lectures start, he just sits in his bed half asleep* (Mother of boy, 19 years; no diagnosis)
Motivation/energy	*Great difficulties motivating herself to studying alone and boosting enough energy. Difficulties getting started* (Mother of girl, 18 years; ADHD and ASD)
Inattention	*Loses focus, gets easily distracted by other things* (Mother of girl, 16 years; no diagnosis)
Depression/anxiety	*The lack of social contact has made my child depressed, and he has lost interest in a lot of things* (Mother of boy, 18 years; no diagnosis)
Digital media	*I have discovered that he is doing other things [e.g., playing games and music] while listening to the teacher, which would not be possible in the classroom* (Mother of boy, 17 years; no diagnosis)
Other child problems	*My son does not feel good at all now when the school is closed* (Father of boy, 16 years; ADHD) *My child has very poor executive abilities. Being in school with good routines helps him with this—now he is just barely present at the online lectures but not more than that* (Mother of boy, 16 years; ADHD & ASD)
**Support (26%)**	
Teachers to child	*It is difficult to get support from the teacher in the same way as during a regular lecture. He has to wait until the teacher answers* via *e-mail, and then he has already lost his focus* (Mother of boy, 16 years old; ADHD) *During regular classes, the teacher can see who needs help. At home, my daughter needs to take the initiative herself to get help* (Mother of girl, 18 years; no diagnosis)
Parents to child	*Need for active support from parents several hours each day* (Mother of girl, 16 years; ADHD) *We are so lucky that I can work from home now during the pandemic, because he would never have managed to do his schoolwork from home without my help* (Mother of boy, 16 years; ADHD and ASD)
School to parents	*It is difficult for me as a parent to support my child, because I do not know what is expected of him. I do not get this type of information from my child* (Mother of boy, 16 years; ADHD)
Extra support/SEN	*Extra support has been canceled or has been very limited* (Mother of girl, 16 years; ADHD)
**Social (18%)**	
General	*Our child thinks the social situation with the schools being closed is a disaster—she really hates being social isolated* (Mother of girl, 17 years; no diagnosis)
Peers	*My daughter feels isolated and misses her friends* (Father of girl, 17 years; no diagnosis)
Family	*We as parents are worried about the situation and we therefore argue more with our child, which leads to more family conflicts* (Mother of boy, 17 years; no diagnosis)
**Home environment (10%)**	
Food	*It takes a lot of time to fix food. He does not even heat food in the microwave oven by himself* (Mother of boy, 17 years; ADHD and ASD)
Exercise/fresh air	*No transportation to school and no movement between classes is now needed so he gets very little exercise these days* (Mother of boy, 17 years; no diagnosis) *He does not get any exercise at all now during school closures [he does not even set foot outside the house for days] so no wonder he cannot pay attention during online teaching* (Mother of boy, 17 years; ADHD)
Space	*She has to sit in the kitchen in our small apartment and gets distracted when I move around* (Mother of girl, 18 years; ADHD)
**Technical problems (9%)**	
Internet connection	*There are problems with the internet connection for some of his classmates—makes it difficult when they are instructed to work together with an assignment* (Mother of boy, 16 years; no diagnosis)
Teaching platform	*Many new digital tools to learn how to handle. Quick adjustments necessary, which means that the different parts have not been coordinated* (Mother of boy, 17 years; ADHD)

**Table 3:** Results of the study for negative effects showing themes, subthemes, and quotes.

Themes/Subthemes	Quotes
**Child factors (38%)**	
Inattention	*Total stimuli reduction at home and all energy can be put into the schoolwork. The entire school day therefore works so much better now when he can focus* (Mother of boy, 17 years; ADHD and ASD)
Mental health	*Much less stress. She is happier and more social* (Mother of girl, 16 years; no diagnosis)
Higher attendance	*It is possible to attend despite having a bit of a cold* (Mother of boy, 16 years; no diagnosis) *He has participated in online teaching, he is asking questions and completing assignments. Things that he has not been doing for several years* (Mother of boy, 17 years; ADHD and ASD)
Responsibilities/skills	*She is learning to be more independent and organizing her schoolwork, increased digital knowledge, adapting to new situations—these are all really important skills for having a successful career later in in life* (Mother of girl, 16 years; no diagnosis)
**Home environment (37%)**	
Transportation	*She does not have to spend lots of time on commuting to and from school every day—time that can now be spent on other things that make her happy and less stressed* (Mother of girl, 16 years; ADHD and ASD)
Sleep	*Does not have to get up as early—everything is working much better [schoolwork, fewer mood swings] now when he finally gets enough sleep every night* (Mother of son, 18 years; ASD)
General	*My child likes it better at home where it is calm and he has all his things around him—he likes that he can use his big computer screen and sit in his comfortable chair while doing his schoolwork* (Mother of boy, 17 years; ASD)
Food	*Will not eat the food that is served at school, so it is much better now when I am working from home and can make sure that he gets a good lunch* (Mother of boy, 16 years; ADHD and ASD)
Exercise/fresh air	*My child and I exercise together now when we are both at home—we go running or take walks. One afternoon we played tennis* (Mother of girl, 16 years; no diagnosis)
**Teaching (15%)**	
Flexibility/independent needs	*They can work more in their own pace* (Mother of son, 16 years; no diagnosis) *It is good that my son can better adapt his schoolwork depending on how tired he is that day* (Father of son, 18 years; ADHD)
Structure/efficiency	*Shorter school days [no recess, no extra time to move between classes] and clearer demands from teachers make the school day more efficient* (Mother of girl, 16 years, no diagnosis)
New methods	*The school has introduced some good new teaching methods that my child really likes. For example, private chats with the teachers have increased opportunities for good communication compared to the regular classroom. The school has discussed the idea that they should keep offering this even after the pandemic. Good for my son as he has difficulties asking questions when other students might hear him* (Mother of boy, 16 years; ASD)
Quality	*Homeschooling is fairer for the students. In the classroom, the teachers have some students they like more. Now there is more focus on the actual assignments and less time for unequal treatment of students* (Mother of boy, 17 years; no diagnosis)
Alternative for student with absenteeism	*Only good things with this! My son answers his teacher when she calls, he asks me if he needs help, but most time he can now managed on his own after being absent from school for almost two years* (Mother of boy, 18 years; ADHD)
**Social (14%)**	
Avoid peers	*The environment here at home is simpler as it does not focus on social interaction in groups, body language, clothes and how people look* (Mother of boy, 17 years; ADHD and ASD)
Family	*Normally, we get maybe 10 min of his time during school days. During distance learning, he has his meals with us several times a day and participates in discussions and watches movies with us—normally he does not have the energy to do this* (Mother of boy, 18 years; ASD)
Isolation/virus safety	*When you have a family member in a risk group for COVID-19 and the whole family needs to be in quarantine, you do not want your child to go to school. Distance learning has therefore been very good for us* (Mother of girl, 16 years; no diagnosis)
Digital interaction	*The whole class has digital meetings with the camera on [if they like] so that they can see each other and this creates a good sense of belonging* (Mother of girl, 17 years; ADHD and ASD)
**Support (7%)**	
Parental engagement	*As a parent, it is fun to get insights into how her teaching is conducted. This is something that we parents do not know much about, especially not now during high school* (Mother of girl, 17 years; no diagnosis)
Parents	*I know so well what difficulties he has, and I can therefore help him and adapt some of the assignments—otherwise he will just give up and won’t do any work at all* (Mother of son, 17 years; ADHD)
Teachers	*The teachers have adapted the teaching, and they are available in the chat function whenever someone needs assistance* (Mother of boy, 17 years; ADHD and ASD)

## Data Availability

Dataset available on request from the authors.

## Correction Statement

Following the removal of this article from the Special Issue “The Impact of Parental Mental Health on Children”, an Editorial Board member reviewed the entire editorial process and confirmed all elements had been carried out as per MDPI Editorial Process policy (https://www.mdpi.com/editorial_process). As a result, the original Academic Editor listed on this publication has been replaced with that of the Editorial Board member who conducted this review. The original publication has now been updated accordingly. This information has also been transmitted to relevant indexing organizations.
